# LINA’s testing infrastructure enables AI to take-off in unmanned aerial vehicles (UAVs)

**DOI:** 10.3389/frobt.2026.1764248

**Published:** 2026-03-13

**Authors:** Hella A. Bolck, Janik Vollenweider, Fabian Merkli, Alexander Barden, Martin Jajcay, Peter Trempeck, Boško Rafailović, Robert Fraefel, Peter M. Lenhart, Ricardo Chavarriaga, Manuel Renold, Jasmina Bogojeska, Thilo Stadelmann, Michel Guillaume

**Affiliations:** 1 Centre for Artificial Intelligence, Zurich University of Applied Sciences, Winterthur, Switzerland; 2 School of Business, University of Applied Sciences and Arts Northwestern Switzerland, Olten, Switzerland; 3 Centre for Aviation, Zurich University of Applied Sciences, Winterthur, Switzerland; 4 Robotics and Perception Group, University of Zurich, Zurich, Switzerland; 5 Matternet Operations GmbH, Dübendorf, Switzerland; 6 Skyguide, Swiss Air Navigation Services Ltd, Geneva, Switzerland; 7 European Centre for Living Technology, Venice, Italy

**Keywords:** artificial intelligence, autonomous systems, BVLOS operations, certification, safety, testing, unmanned aerial systems

## Abstract

The development of autonomous aerial robots capable of safely navigating complex real-world environments without or with little human intervention represents a major milestone in robotics and artificial intelligence (AI). While rapid advances in AI-enabled decision-making, sensing, and control systems are unlocking new capabilities for unmanned aerial vehicles (UAVs), their translation into safe and scalable real-life applications remains a major challenge. In this Perspective, we examine key AI technologies relevant to aerial autonomy and discuss early application scenarios in unmanned aviation and airspace management, with a focus on their assurance-relevant properties. We analyze regulatory obstacles that limit deployment, particularly for AI-enabled and beyond visual line of sight (BVLOS) operations, and highlight why traditional risk assessment and certification approaches are need to be updated to account for adaptive, data-driven systems. Building on this analysis, we argue that testing infrastructure must be understood as a core scientific instrument, enabling systematic evidence generation under realistic and safety-critical conditions, validating autonomous functions, ensuring safety, and building trust among regulators and the public. As a concrete example, we introduce LINA, a scientifically-grounded, integrated experimentation and validation platform in Switzerland designed to support iterative, regulator-aware development of autonomous systems across technology readiness levels. We highlight how LINA function as sandbox for system-level science, regulatory learning, and trust building, thereby enabling the responsible and societally acceptable integration of autonomous aerial systems and strengthening Switzerland’s role in advancing aerial robotics research and innovation.

## 1 Introduction

Unmanned aerial systems (UAS) are revolutionizing the airspace, offering unprecedented opportunities across diverse sectors such as logistics, mobility, public safety, agriculture, and disaster management. Equipped with advanced sensors and increasingly autonomous capabilities, drones can navigate complex environments, operate beyond visual line of sight (BVLOS), and perform tasks only recently considered impossible for flying robots (Petersen et al., 2019; Floreano and Wood, 2015; Falanga et al., 2020). The integration of artificial intelligence (AI) is a key enabler of this development supporting autonomous decision-making, improved airspace management, and increasingly complex operational behaviours (Li et al., 2023). In the aviation domain, however, the integration of AI raises challenges that go beyond technical performance. Stringent safety and certification requirements, accountability, and the need for explainability in safety-critical decision-making impose constraints and as a result a fundamental gap emerges between AI capabilities demonstrated in research settings and their real-world readiness in regulated airspace. This gap is amplified by a structural mismatch in development cycles as AI has been advancing rapidly over short timeframes within months, whereas aviation certification and regulatory adaptation typically unfold over many years. At the same time, engineering innovation, particuarly in robotic aplications fundamentally depend on iterative development, rapid experimentation, and frequent feedback between design, testing, and deployment. Bridging these timelines therefore requires approaches that enable fast, repeatable evidence generation while remaining aligned with safety and regulatory expectations. From a scientific perspective, this raises central questions: how can non-determinisitic, adaptive, data-driven AI systems be systematically validated, understood, and trusted, and how can evidence be generated to support regulatory approval and operational acceptance? Addressing these questions requires more than improved algorithms alone. It necessitates appropriate tools, including explainable AI (XAI), structured testing and validation methodologies, and large-scale experimentation environments capable of generating evidence under realistic operational conditions. Switzerland, a leader in enigineering and AI research and development, is well-positioned to contribute to progress in these areas (Christen et al., 2018).

Building on this context, in this Perspective article, we synthesize insights from AI research, aviation regulation, and testing practice to identify structural bottlenecks and outline how they can be addressed. We advance an infrastructure-based perspective toward the safe deployment of autonomous aerial systems, with a particular focus on the instrumental layer required to translate AI innovation into operational reality. Central to this discsussion is LINA (Shared Large-Scale Infrastructure for the Development and Safe Testing of Autonomous Systems^1^), an academic-industry collaboration, that provides the necessary facilities, processes, and expertise to systematically test, interpret, and validate autonomous systems under realistic and regulator-aware conditions.

While much of the existing literature explores technical innovations in AI or specific UAV applications (Hanover et al., 2024; Palossi et al., 2022; Caballero-Martin et al., 2024; Firoozi et al., 2025), comparatively little attention has been paid to the additional tools and facilities required to facilitate their societal integration, or to how these shape the development, validation, and use of AI systems themselves. In Section 2 of this Perspective, we review key applications of AI that are central to advancing UAS autonomy and discuss their implications for safety assurance and operational readiness. Section 3 highlights application scenarios piloting AI in unmanned aviation. This is followed by a discussion of the regulatory challenges associated with deploying AI-based UAVs in real-world settings in Section 4, where high levels of autonomy and adaptive behavior raise critical questions around safety assurance, accountability, and risk assessment. In this context, we consider how concepts like explainable AI (XAI) can contribute to transparency and trust (Xu et al., 2019). Finally, in Section 5, we reflect on our experience with building LINA to illustrate how dedicated testing infrastructures function as scientific instruments that bridges the gap between AI research and operational deployment. By doing so, this article contributes to the scientific discourse on AI and robotics clarifying the infrastructural conditions under which autonomous systems can move from experimental capability to real-world application.

## 2 AI for UAS: gaps towards full integration

UAS have evolved to advanced systems capable of complex missions. Yet, most remain only partially autonomous, relying on a human for critical decisions (Caballero-Martin et al., 2024). Achieving higher levels of autonomy, as defined in AI and engineering (Tallam, 2025; Formosa et al., 2025; Lenhard, 2024), where UAS can navigate, make decisions, and adapt without human input, represents a significant technological challenge (Elmokadem and Savkin, 2021). However, advances in AI are steadily bringing this goal closer (Kaufmann et al., 2023; Azar et al., 2021). Specifically, four areas of AI stand out with respect to their utility for UAS and will be introduced with an overview of their prospects and challenges below: Reinforcement learning (RL) and model predictive control (MPC) as methods to control the UAV; computer vision (CV) methods to enhance its sensing and perception capabilities; and integration with large language (LLMs) and other foundation models (Firoozi et al., 2025; Kawaharazuka et al., 2025) that hold the potential to enhance contextual understanding and decision-making further (Javaid et al., 2024). Today most UAS operate within a “human in the loop” framework (Agrawal and Cleland-Huang, 2021; Mussi et al., 2025). While they are capable of performing tasks such as following predefined routes, maintaining stable flight, and returning to base autonomously, human intervention remains essential for managing unexpected events. Achieving full autonomy so that a single human operator will be able to handle not only one drone but to manage a fleet would require the UAV to sense its surroundings, dynamically plan flight paths, and respond to sudden obstacles in real-time (Anicho and Cvetković, 2023). Although prototypes of such autonomous systems exist, achieving reliable and certifiable operations remains a scenario of the future, contingent on demonstrating fitness for purpose under realistic operational conditions (Elmokadem and Savkin, 2021; Nawaz, 2024).

A key hurdle is enabling UAVs to navigate and control their flight paths in dynamic environments. RL, the branch of machine learning (ML) aimed at learning to act purely based on rewards obtained through interaction with a particular environment (Tang et al., 2025), has shown considerable promise. RL enables UAS to acquire control and learn optimal behaviours through trial and error in simulated environments (Song et al., 2023) which can subsequently be transferred to the real-world settings. Through repeated iterations, RL agents refine their learned policies to adapt to changing conditions and unforeseen circumstances (Sadeghi and Levine, 2017; Aljalbout et al., 2024). However, RL is typically sample inefficient, requiring a very large number of environment interactions to achieve reliable performance. This leads to extensive training demands and, together with the often limited interpretability of learned policies, poses significant challenges for deployment in safety-critical aviation contexts (Haarnoja et al., 2018). Its opaque decision-making also creates challenges for regulatory approval, as transparency and explainability are key requirements for safety and trust. Consequently, a substantial real-world readiness gap remains for RL from both pragmatic and regulatory perspectives. At the same time, RL remains one of the most promising approaches for enabling complex, adaptive behaviour in UAS. Bridging this gap requires large-scale experimentation facilities capable of generating realistic data and providing comprehensive insight into system behaviour.

In contrast to RL, MPC employs an explicit, model-based optimization framework in which a mathematical representation of the UAS dynamics and constraints is used to predict future system trajectories over a finite receding horizon (Krinner et al., 2024). At each control step, an optimal control sequence is computed by solving a constrained optimization problem, of which only the first control input is applied before the horizon is shifted forward. This explicit handling of system constraints and safety envelopes enhances interpretability and traceability, making MPC generally more amenable to regulatory oversight and certification processes than data-driven RL approaches. However, MPC faces notable challenges for safe societal integration. Its reliance on accurate system models makes performance sensitive to modeling errors, unmodeled dynamics, and environmental uncertainties, which can significantly degrade behaviour outside nominal operating conditions. Additionally, solving an optimization problem online imposes significant computational demands, particularly for high-dimensional and non-linear UAS models operating under real-time constraints. These factors contribute to MPC’s deployment readiness gap, wherein controllers that perform well in simulation or controlled environments struggle to scale robustly to operational environments with limited onboard computing resources and rapidly changing conditions (Song et al., 2023; Schwenzer et al., 2021). The choice between RL and MPC, or the adoption of hybrid approaches that combine learning-based adaptability with model-based safety guarantees, therefore strongly depends on specific mission requirements and regulatory considerations and must ultimately be informed by systematic experimental validation under realistic operational conditions.

As a third AI aspect, CV has become central to enable UAVs to perceive and interpret their environment. CV methods evolved rapidly from a primary academic topic to frequently applied, practical tools during the 2010s (Stadelmann et al., 2019). With comarativel modest hardware requirements by today’s standards, many CV algorithms can now operate in real time on board of UAS platforms. Hence, while GPS and inertial sensors can provide basic navigation, they lack the contextual awareness required for complex operations, whereas CV systems equipped with object detection algorithms enable real-time identification of obstacles, terrain, and other air traffic (Arafat et al., 2023). Capabilities that are increasingly mandated by regulatory frameworks through detect-and-avoid requirements. However, the fundamentally statistical way in which CV methods process visual data makes them susceptible to errors that humans would not typically make (Kurakin et al., 2018; Stadelmann, 2025). As a mitigation, Multimodal LLMs (MLLMs) could add an additional layer of intelligence to UAS, enabling a richer contextual understanding of the sensory input over time.

In principle, such models could help filter implausible detections, reduce brittle failure modes, and even issue control commands as part of integrated vision–language–action (VLA) architectures (Kawaharazuka et al., 2025; Roost et al., 2020). Beyond natural-language interaction with human operators, a UAS equipped with vision sensors and an LLM could analyze aerial imagery, combine it with domain-specific knowledge, and generate actionable insights. In agriculture, for example, such systems could assess crop health following rainfall and recommend targeted interventions, including precise fertilizer application (Zhong et al., 2024; Zhu et al., 2024). Nevertheless, as with other ML approaches discussed in this section, foundation models like (M)LLMs and VLAs lack an explicit causal understanding of the world limiting their ability to reliably relate observations or predicted control actions to downstream consequences (Mai et al., 2024). In the context of autonomous physical systems, world models can offer a promising direction by explicitly learning cause–effect relationships from data (Matsuo et al., 2022). Closing the operational and societal integration gap for CV-based autonomy therefore requires careful trade-offs between error risks, practical considerations like real-time constraints, and advances beyond the current state of the art in world modeling (Tuggener et al., 2022; Balestriero and LeCun, 2025). These trade-offs must be informed by evidence generated through large-scale experimentation in operationally representative conditions, rather than through compartmentalized small-scale laboratory studies alone.

In summary, despite significant progress towards autonomous UAS, many obstacles remain. Addressing these challenges and ensuring reliable responses also in edge cases is essential to prevent accidents. Furthermore, transparent, explainable AI models are an indispensable aspect to build such trust and ensure safety (Denzel et al., 2024). Most importantly, further developing suitable AI systems and integrating them in UAS will require collaboration among researchers, engineers, regulators, and industry stakeholders and depends on access to dedicated testing infrastructures that enable rigorous, iterative, and regulator-aware experimentation under operationally representative conditions.

## 3 AI use cases in unmanned aviation

Nevertheless, AI is already becoming a cornerstone for automating essential tasks such as navigation, object detection and mission planning (Telli et al., 2023; Sai et al., 2023). Moreover, concrete initiatives already illustrate both the potential of AI-enabled UAS and the challenges of their integration into public airspace. In Switzerland, Dufour Aerospace and Air Zermatt are jointly exploring the use of drones to support emergency services in rugged mountain terrain. Their multi-year program investigates scenarios ranging from medical supply transport during disrupted road access to terrain analysis and coordinated operations between helicopters, drones, and other airspace users in the Swiss Alps (Trailblazing, 2025). Similarly, in the healthcare domain, LINA consortium member Matternet pioneers time-critical transport of laboratory samples and medical supplies, which has emerged as one of the most promising applications, where on-demand drone delivery can significantly accelerate diagnostics and improve patient care (Matternet, 2025). Both examples demonstrate that such use cases can only move beyond technical feasibility once the risks to individual safety and broader societal impact have been systematically assessed and shown to be acceptable.

In this context, sense-and-avoid (SAA) capabilities are widely regarded as pivotal for the safe and scalable integration of UAS into existing airspace (Fasano et al., 2016; Yu and Zhang, 2015). Advanced SAA systems promise to significantly reduce or eliminate the need for human intervention, enabling UAVs to complete missions independently, even without reliance on satellite or ground communication (Mishra and Palanisamy, 2023). These systems typically combine sophisticated AI-based perception and decision-making with a diverse range of onboard sensors including radar, LiDAR, ADS-B, optical and infrared cameras (Rezwan and Choi, 2022). While SAA systems perform impressively in controlled environments (c.f. Section 2), their reliable operation in complex, uncontrolled airspace remains a major challenge. A key distinction in this context is between cooperative and non-cooperative airspace users. In controlled or semi-controlled airspace, cooperative traffic, equipped with transponders or ADS-B, actively broadcasts its position, simplifying conflict detection and resolution (Fasano et al., 2016). In contrast, uncontrolled airspace often include numerous non-cooperative actors such as paragliders, birds or hobby drones, which do not emit positional data. Detecting and avoiding them requires heavy reliance on passive sensing modalities, including optical cameras, radar, or acoustic sensors, combined with AI-driven object detection and predictive modeling. This distinction fundamentally shapes the design, validation, and certification requirements of SAA systems and underscores why performance demonstrated in cooperative scenarios does not directly translate to operational readiness in mixed traffic environments (Fasano et al., 2016). This gap closely aligns with current regulatory concerns articulated by the European Union Aviation Safety Agency (EASA). In its recently released NPA 2025-07, EASA explicitly identifies non-deterministic and adaptive AI behaviour as a key assurance challenge, emphasizing that trustworthiness must be demonstrated through evidence generated under representative operational conditions, rather than through design-time verification alone (NPA, 2025-07, 2025).

To support more complex tasks, AI-based UAS must be able to cope with highly variable and at times uncertain conditions resulting from mixed traffic, unpredictable weather, and dynamic environments, all within the strict requirements of safety-critical aviation systems. Yet, current flight-testing regulations offer limited flexibility for experimentation or scaling of emerging technologies as they often impose restrictive operational constraints (Rea, 2024). This warrants closer examination, particularly given that human operators are not inherently better suited to handle these multifaceted requirements. In this context, AI emerges as a compelling solution for addressing the increasingly intricate demands in modern aviation. Fan et al. (2020) even argue that AI is particularly well-positioned to address the interconnected tasks of (i) intelligent perception, (ii) reasoning, and (iii) decision-making, core components of autonomous flights and key sub-tasks of SAA systems. While some AI-driven capabilities are already in use today, integrating more advanced functionalities is expected to broaden the range of missions significantly, including extended-range operations (Telli et al., 2023; European Union Avia tion Safety Agency, 2023). Additionally, AI-based SAA could reduce operational costs by replacing current risk mitigation measures, such as additional observers or highly restrictive flight paths once their safety has been rigorously validated and is broadly accepted.

AI and aviation also intersect prominently when it comes to the future of air traffic management. The full integration of UAS into shared airspace will require robust traffic management based on Unmanned Traffic Management (UTM) or U-Space services and technologies that act as an extension to traditional Air Traffic Control (ATC) that is currently used for managing the safe and orderly flow of manned aircrafts. Future air traffic management systems must coordinate large numbers of manned and unmanned vehicles simultaneously, ensuring safety and efficiency. This coordination depends not only on digital infrastructure and AI-based decision logic, but also on the physical sensing and communication equipment installed on each aircraft as well as on the interoperability between these systems. Fan et al. refer to the innovation push of U-Space services as “digitization of the operational space” emphasizing the need for coordinated advances on both the UTM and ATC side (Fan et al., 2020). Accordingly, the U-Space framework is envisioned to provide services such as flight authorization, tactical conflict resolution, and dynamic capacity management (EASA, 2024a; EUROCONTROL, 2025). Importantly, these developments must align with the position of EASA, frameing AI trustworthiness as a socio-technical property encompassing not only algorithms, but also human interaction, operational procedures, and the broader societal context (NPA, 2025-07, 2025). U-Space services are intended to be delivered through a commercial environment with implementation progressing incrementally and subject to regular monitoring, following the initial blueprint introduced in 2017 (EUROCONTROL, 2025; Joint Undertaking, 2017). However, as of today, U-Space services remain in an early stage of maturity, with full-scale implementation unlikely in the near term, underscoring the substantial technological and regulatory challenges that must still be addressed.

In any regard, according to EASA, AI is expected to become indispensable in aviation, particularly for traffic management and the safe coexistence of manned and unmanned aircrafts (European Union Avia tion Safety Agency, 2023). Given the safety-critical nature of these functions and the early maturity of advanced U-Space services, their development and deployment depend on large-scale testing and validation infrastructures capable of generating evidence under operationally representative conditions, thereby supporting their eventual integration into public airspace (Joint Undertaking, 2017).

Recent developments in Generative AI (GenAI) and LLMs further expand the potential for AI in aviation. For instance, LLMs have shown promise in incident report classification (Tikayat Ray et al., 2023). Private companies like IBM are exploring the potential of GenAI predominantly in manned aviation for applications such as personalizing pilot training, optimizing flight routes, supporting predictive maintenance and many more (IBM, 2025). Also unmanned aviation can greatly benefit from GenAI, as it could be used to streamline key processes, such as automating elements of the flight approval process and particularly the Specific Operations Risk Assessment (SORA) methodology. GenAI could, for example, assist in identifying risks and finding mitigation actions through relevant regulatory compliant measures and thus easing the intense bureaucratic burden for commercial drone operators. From a regulatory perspective, such AI-supported processes must remain transparent and auditable, as their outputs influence operational or safety-related decisions (NPA, 2025-07, 2025). Beyond SORA, the application of GenAI is much broader. It can enhance UTM services by optimizing flight routes based on flight plans and synthesizing data where its real-life generation is not feasible (Liu et al., 2024; Sun et al., 2024).

Thus, early use cases already foreshadow the transformative potential of AI to improve the safety and efficiency of UAS operations. However, to overcome remaining challenges and fully realize the benefits of AI in aerial robotics, it is crucial to rigorously investigate these technologies in secure and representative environments that allow systematic testing, validation, and certification-readiness (Christen et al., 2018; Rea, 2024). Accumulating flight evidence with AI-enabled UAS will not only enhance their reliability and scalability but also establishing a foundation for future advancements in areas such as delivery, urban air mobility and even manned aviation.

Beyond enabling individual use cases, accessible large-scale testing infrastructures are a prerequisite for generating comparable and regulator-relevant evidence, enabling academia–industry collaboration, applied research, and iterative co-development cycles that are essential for maturing AI-enabled UAS capabilities. This need is particularly acute for BVLOS operations, where the absence of harmonized test-area standards currently limits the comparability and transferability of evidence across sites and jurisdictions. Recent regulatory developments further underscore this requirement by emphasizing that assurance of AI-based systems must be grounded in evidence generated under representative operational conditions, addressing non-determinism, learning effects, and system-level interactions throughout the system lifecycle (NPA, 2025-07, 2025). In this context, initiatives such as LINA directly address emerging scientific, technological, and regulatory expectations by providing a framework in which AI-enabled UAS capabilities can be systematically observed and matured toward safe and societally acceptable deployment.

## 4 Risk and safety assessment requirements for AI-Based UAS

**FIGURE 1:**
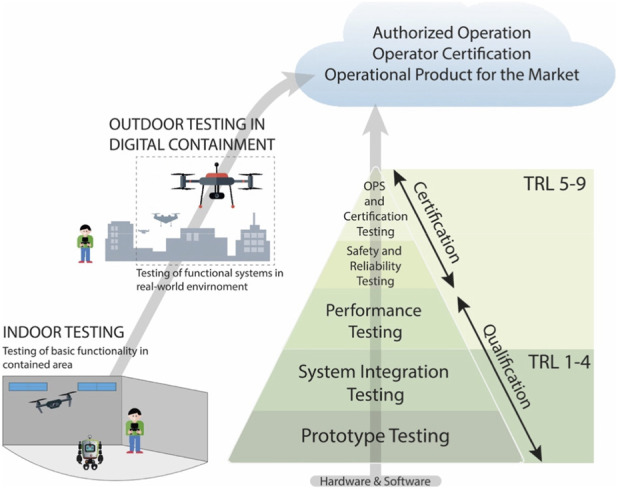
Overview of the testing and certification pathway for autonomous UAS, moving from early-stage prototype testing (TRL 1–4) to system-level validation (TRL 5–9) and ultimately to deployment and market-readiness. In LINA, we offer indoor testing facilities to verify core functionality and integrating subsystems. As technologies mature, testing can shift to outdoor sites featuring digital containment strategies to evaluate safety, reliability, and performance under real-world conditions.

Any UAS, built with or without AI components, needs to undergo thorough risk and safety assessment before it can be used around humans (Ariante and Del Core, 2025). Most applications are initially tested under laboratory conditions in controlled indoor settings devoid of weather interferences and without regulatory restrictions. In this phase, engineers can rapidly improve state estimation, obstacle avoidance, and trajectory planning. To achieve this, they often use motion capture systems for precise ground-truth data (Song et al., 2023). For more advanced tests, wind and other weather conditions can be simulated. However, indoor facilities are often limited in size, cannot replicate real-world complexity and GPS signals are frequently unavailable. At later Technology Readiness Level (TRL) stages, outdoor testing therefore ensures the system’s resilience under varied conditions (Figure 1). However, outdoor testing most often requires adherence to strict safety protocols and regulatory frameworks (EUR-Lex, 2025). This applies not only to autonomous UAS but also to automatic and manually controlled systems. UAS outdoor operations can be carried out in one of three categories: open, specific, and certified (EUR-Lex, 2025). The open category is intended for low-risk operations, allowing activities within predefined safety limits without requiring prior authorization. These include hobbyist flights and basic commercial operations, provided they adhere to strict operational limits. More complex operations including autonomous flights can only be performed in the specific category that is designed for medium-to high-risk operations (EASA Artificial Intelligence Roadmap 2, 2023). In this category, a SORA is mandatory to obtain approval from national aviation authorities such as the Swiss Federal Office of Civil Aviation (FOCA). The SORA assesses the ground risk, i.e., the potential impact on individuals and property, and the air risk, which addresses the likelihood of mid-air collisions involving both manned and unmanned aircraft. To mitigate these risks, operators must fulfill Operational Safety Objectives (OSOs), that address the technical design of the UAS, crew competency, human error and various operational conditions. Finally, the certified category covers operations of similar complexity as manned aviation. Examples are air taxiing services or military reconnaissance missions. Autonomous operations are currently restricted to the specific or certified categories due to their higher complexity and risk (EASA Artificial Intelligence Roadmap 2, 2023).

Technologically, UAS are already capable of flying long distances without human eye contact (BVLOS) using a combination of GPS navigation, onboard sensors, and AI for obstacle avoidance. Companies have demonstrated autonomous deliveries across urban areas, infrastructure inspections along pipelines, and even urban air taxi prototypes navigating complex routes. However, regulatory readiness lags behind. Existing regulations do not yet explicitly account for embedded AI systems, highlighting the need for updated guidelines tailored to these technological advancements. Regulatory approaches currently vary across jurisdictions, with some establishing dedicated frameworks for AI systems (e.g., the EU AI Act), which means that UAS developers must often simultaneously comply with both domain-specific and AI-specific requirements. While the integration of AI in the aviation domain may not demand entirely new risk assessment methodologies, it does require appropriate enhancements to existing frameworks. EASA is currently working on establishing such standards through its “Artificial Intelligence Roadmap 2.0”, which outlines the basis for the safe integration of AI into aviation while ensuring safety and trust (EASA Artificial Intelligence Roadmap 2, 2023). The roadmap categorizes AI systems into three levels based on autonomy and authority. Level 1 refers to AI systems that assist human operators, level 2 incorporates human-AI collaboration, and level 3 is for fully autonomous systems. The concept paper on level 1 and 2 ML applications emphasizes the development of standards, methods, and regulations for certifying AI systems, with a focus on transparency, reliability, and maintaining risk levels equivalent to those of non-AI systems (EASA, 2024b). It also estimates that AI systems up to Level 2 could be certified according to the current state of AI technology, while further advancements are necessary to enable certification of higher autonomy levels.

Taken together, it is increasingly cleat that the complex behaviours enabled by AI integration into UAS significantly complicate the assessment of regulatory compliance. In particular, the non-deterministic and data-dependent behaviour inherent to many modern AI methods used for control, perception, or decision-making can lead to unclear or unpredictable performance in edge cases, thereby increasing the demands on safety assurance. This poses a fundamental challenge to established aviation certification practices, which are traditionally grounded in demonstrating extremely low probabilities of catastrophic failure (on the order of 10^-9^ per flight hour) through deterministic system analysis and formal verification. For AI-enabled systems, such guarantees are difficult to establish, as learning-based components often resist exhaustive formal methods and instead require probabilistic validation approaches based on empirical evidence. While such approaches can characterize typical performance, they may fail to capture rare but safety-critical failure modes that dominate aviation risk assessments. In addition, assessing how an AI-driven system balances mission objectives against safety constraints is further complicated by opaque algorithmic behaviour and limited explainability, reducing traceability and hindering confidence in compliance with safety objectives.

To address challenges from complex AI behavior and ensure their safe and effective operation in diverse environments, risk assessment must extend beyond traditional system verification. It should include explicit documentation of all AI components (AI models, their specific tasks, training data) and their operational context such as their interaction with sensors, and external data inputs to make decisions. Systematic testing across diverse and adverse scenarios, and continuous monitoring after deployment will capture learning effects and performance drift. Appropriate strategies should prioritize explainable AI tools to enhance transparency and support human oversight. Implementing fail-safe protocols that entail redundancy, and fallback systems can handle AI failures similarly to human errors and systems that continuously update based on risk monitoring and assessment contribute to safe and reliable operation (Lingam et al., 2025). Future certification frameworks must also address AI trustworthiness as an operational property, requiring the translation of trustworthiness-based frameworks into actionable certification schemas that combine procedural and technical evaluation (NPA, 2025-07, 2025; Weng et al., 2024; Frischknecht-Gruber et al., 2025). These requirements go beyond algorithmic performance and reinforce the need for experimentation environments capable of supporting evidence generation across the full operational lifecycle, particularly for increasingly autonomous UAS. This shifts the focus from abstract regulatory principles to the concrete question of how such evidence can be generated in practice.

## 5 LINA is a unique integrated sandbox for testing, validation, and regulatory learning

Rigorous testing and validation emerge as a central mechanisms for establishing safety, trust, and operational acceptance in autonomous aerial robotics. This demands structured, repeatable, and regulator-aware experimentation that allows complex systems to be observed, compared, and matured across their full development lifecycle. In public airspace, autonomous systems cannot simply “be flown”; instead, they must be developed within state-of-the-art sandbox environments that enable controlled risk exposure, evidence generation, and iterative refinement under expert guidance.

**FIGURE 2:**
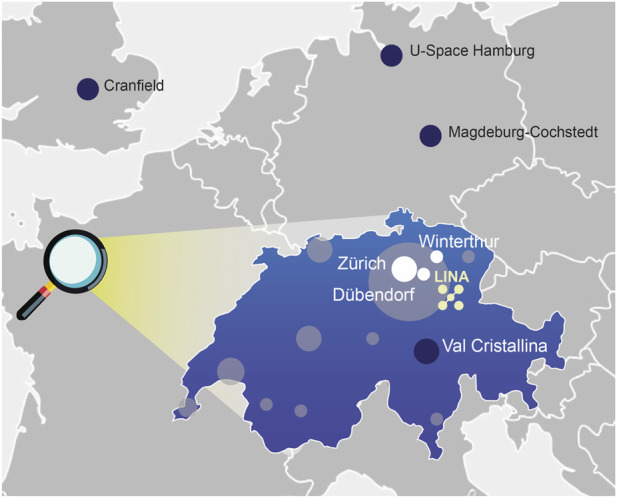
Selected test sites for aerial robotics and autonomous systems across Middle-Europe, highlighting the LINA Infrastructure in the Greater Zurich Area including relevant cities (white). Switzerland is magnified within Europe, with the country’s key hubs of the current drone industry shaded in grey (based on data form Drone IndustryInsights UG, 2024). Maps sourced Free Vector Map.

Switzerland is recognized as a global leader in AI and robotics innovation. Nevertheless, a dedicated test infrastructure for UAS and autonomous aerial robotics supporting the entire development pipeline has long been missing (Christen et al., 2018; Drone Industry Insights UG, 2024). The need for such an infrastructure was first highlighted in 2018, when a TA-Swiss study (Christen et al., 2018) called for collaborative action between the Swiss federal government, academia and industry stakeholders to establish a national UAS testing area. This gap has since widened, as confirmed by industry stakeholders (Drone Industry Insights UG, 2024), who continue to report limited access to suitable, scalable, and regulatorily integrated test environments. LINA was established in response to this structural deficit as a framework that integrates infrastructure, expertise, and regulatory alignment. By providing a continuous pathway from early prototyping to deployment-relevant validation (Figure 2), LINA eliminates the fragmentation that typically occurs between laboratory experiments, pilot trials, and operational testing. In addition to developing and examining advanced AI algorthms, LINA supports evidence-based safety and certification research. It enables reproducible experiments, comparable datasets, and shared evaluation protocols that directly address regulatory questions around trustworthiness, non-determinism, and system-level assurance.

This is achieved through repeated experimentation under consistent instrumentation, operational constraints, and safety procedures (i.e., shared evaluation protocols), leading to uniform (i.e., comparable) datasets, allowing results to be generated under comparable conditions. In this way, LINA creates the foundation for the emergence of common evaluation criteria and structured experimental evidence for AI-enabled UAS, supporting benchmarking, next-generation algorithm development, and ultimately helping to close the gap between working demonstrations and reliable, deployable systems (i.e., real-world datasets) (Zabihi, 2025). This also makes LINA a space for regulatory learning, where new operational concepts, approval pathways, and airspace integration strategies can be explored collaboratively. Anchored in the Canton of Zurich in Switzerland, LINA is part of a unique ecosystem, positioned in a region that is home to numerous drone and robotics companies as well as renowned institutions (ETHZ, UZH, ZHAW and EMPA) supplying highly skilled engineers and advancing cutting-edge technologies in areas such as software, AI and batteries. Thus, LINA functions as a hub for ecosystem and capability building. By significantly lowering barriers to access and providing shared methods and tooling, it strengthens academia–industry collaboration, supports projects across many application domains, and enables iterative co-development across all technology readiness levels. In this sense, LINA contributes to long-term national capacity building by anchoring expertise, validation competence, and autonomous systems research within Switzerland.

One of LINA’s main objectives is to provide comprehensive infrastructures that serves this broader mission (Figure 1). The indoor testing facilities support early-stage prototypes (TRL 1-4) and high-risk applications, where controlled environments are essential for evaluating system performance without external disturbances like adverse weather condition. Since 2022, LINA has operated multiple indoor facilities before transitioning to a repurposed industrial space in Winterthur, where large, unused halls provide an ideal environment for developing and refining autonomous robotic technologies (Figure 2). This indoor facility is equipped with an advanced motion capture system that enables precise tracking of drones and mobile robots, crucial for algorithm validation, movement analysis, and performance optimization. Additionally, digital twin simulations allow researchers to model, test, and refine autonomous systems virtually before deploying them in physical environments. These capabilities support interdisciplinary research, including human-drone interaction, AI-driven navigation, and collaborative testing of aerial and ground-based robotics.

For most autonomous systems in mid-to late-stage development (TRL 5-9), indoor testing alone is not sufficient as real-world outdoor testing is critical to ensure functionality and reliability. For such needs, we have established the LINA Outdoor Test Range at the Hegmatten Airfield in Winterthur. It supports a wide range of tests and applications, from basic hovering and short-range maneuvers to complex BVLOS operations critical for UAS applications in logistics, emergency response, and surveillance, where direct visual contact with the UAV is not possible. In collaboration with its consortium partner Skyguide, LINA is positioning the site as a testbed for advancing air traffic management solutions like the U-Space in Switzerland (EASA, 2024). Close partnerships with the Swiss FOCA and alignment with EASA standards will further streamline approval processes, expediting the progression from prototype to market-ready technologies. In addition to its core facilities, LINA’s ecosystem is strengthened by key partnerships, such as with the DroneHub at EMPA (Swiss Federal Laboratories for Materials Science and Technology) in Dübendorf. The newly opened NEST complements LINA’s capabilities by enabling testing at the intermediate TRL (TRL 3-9) including navigation between indoor and outdoor environments and testing in challenging conditions, such as extreme heat, water or in-between natural obstacles like trees and urban infrastructure (Kaya et al., 2026).

Several other test sites across Switzerland and Europe offer specialized capabilities but many have limitations in providing the accessible, versatile, and regulatorily integrated infrastructure that users increasingly require (Scott BI and Andritsos, 2023). One of the most advanced outdoor test range in Switzerland is located in Val Cristallina, high in the Swiss Alps. This pioneering site was initiated by Armasuisse Wissenschaft und Technologie (W + T), the civilian research branch of the Swiss military, and is operated in collaboration with RUAG AG. It was the first site in Switzerland to permit BVLOS operations for external users. It is particularly well-suited for high-risk testing, offering a remote and controlled environment with minimal interfering air or ground traffic. However, its isolated alpine setting comes with trade-offs: its remote location, limited availibilty due to weather conditions and dual use as a military shooting range, and complex access and safety protocols significantly hinder iterative testing and agile development cycles. Moreover, as a greenfield site, it lacks the infrastructural context needed for operations that simulate interaction with more urban and populated environments.

An advanced and well recognized facility outside of Switzerland is the Experimental Test Center for Unmanned Aircraft Systems at the Airport Magdeburg-Cochstedt operated by the German Aerospace Center (DLR, 2025) (Figure 2). It supports large-scale UAS research, testing, validation, demonstration, training and networking for science and industry stakeholders (DLR, 2025). There is a primary focus on drone development and system-level testing as well as on complex legal and procedural frameworks. It can accommodate large UAVs including drones for cargo transport. However, it primarily serves well-established stakeholders who already possess the necessary regulatory approvals. External users must navigate complex approval processes independently, making access challenging. Additionally, its remote location presents logistical hurdles.

The U-Space Reallabor Hamburg was another German flagship initiatives and part of a broader strategy for developing smart-city infrastructure and sustainable transport solutions. Covering approximately 10 km^2^ over the Port of Hamburg, the Reallabor focussed on testing air traffic management, communication systems, and public acceptance models (BMV, 2025). Running from May 2021 to November 2022, the pilot demonstrated how drones can be safely integrated into existing air traffic structures and provided a blueprint for broader U-Space implementation in Germany and Europe. While the Reallabor successfully validated specific use cases and services, it was designed as a temporary demonstration environment, not as a long-term, structured and scalable testing infrastructure for diverse autonomous systems.

The Cranfield Air Corridor in the United Kingdom (United Kingdom) also enables testing of unmanned and manned aircraft in shared airspaces leveraging Cranfield University’s aerospace expertise and close collaboration with the United Kingdom Civil Aviation Authority (Cranfieldairport, 2025). It allows for safe, controlled trials of BVLOS operations and autonomous aviation systems supporting a variety of projects aimed at accelerating the adoption of autonomous technologies and facilitating airspace integration. Its focus is largely on aviation research, air mobility and the broader air traffic management ecosystem.

Various smaller European initiatives exist, each with a unique profile. Efforts are underway to harmonize test site capabilities and regulatory sandboxes at the EU level. EUROCONTROL plays a key role in fostering their collaboration and developing a comprehensive capability map (EUROCONTROL, 2023). Within this evolving landscape, LINA stands out as an accessible, flexible, and user-centered platform, uniquely positioned at the heart of Europe close to urban and densely populated settings, where such capabilities are most needed. It supports both early-stage and advanced autonomous system testing, integrates cutting-edge expertise across the whole drvelopment cycle and contains a clear pathway toward regulatory integration and ecosystem engagement, thus setting new standards in autonomous technology development, and distinguishing itself as a unique European infrastructure.

## 6 Conclusions and future recommendations

The remarkable advancements in AI are bringing UAS closer to autonomous operation supported by context-aware decision-making and increasingly natural interaction with human operators. The integration of RL, MPC, real-time object detection, and LLMs promises more adaptive behaviour and intuitive supervision. However, the transition of these technologies from controlled laboratory environments to deployment in dynamic, shared airspace remains a substantial challenge. Achieving higher levels of autonomy requires extensive testing and validation under realistic and safety-critical conditions.

Experience from other domains, most notably autonomous road vehicles, illustrates that technical maturity alone is insufficient when systems must operate in open, unpredictable environments. Similar to UAS operating in uncontrolled airspace with mixed traffic, self-driving cars face rare but safety-critical edge cases that cannot be exhaustively addressed through design-time verification alone. In both domains, safe deployment depends on robust testing infrastructures, explainable and transparent AI behavior, systematic safety assurance, and alignment with evolving regulatory frameworks (Kenny et al., 2024; Santoni de Sio, 2021). At present, the absence of formal guidelines for designating and approving UAS test areas presents a significant barrier to systematic assessment, particularly for AI-enabled systems and BVLOS operations. Regulatory uncertainty regarding acceptable risk levels, mitigation strategies, and certification pathways further constrains the development of complex missions, such as autonomous flights over populated areas, despite their high commercial and societal relevance. Analogous to the role of simulation environments and test tracks for cars (Vishnukumar et al., 2017), dedicated experimentation infrastructures are essential for stepwise, regulator-informed development, validation, and certification of AI-driven UAS. Strucutres like LINA provide the essential platform for stepwise, and regulatorily informed development, validation and certification of the full range of AI-driven UAS systems, ensuring their safe integration into shared (air)spaces.

Building on these insights, several future priorities emerge for advancing AI-enabled UAS toward safe and scalable deployment:

- Certification frameworks should move beyond static approval toward continuous assurance models, explicitly accounting for data dependence, non-deterministic behavior, and post-deployment learning effects. This includes requirements for ongoing monitoring and reporting of operational performance, such as near-misses, overrides, and anomalous behavior, to support regulatory oversight. Responsibility for this should be distributed: operators collect and report operational data, regulators define reporting requirements and oversight mechanisms, and infrastructures such as LINA support the development of monitoring methodologies, unified protocols, and evidence interpretation. Methodological foundations are increasingly developing in current academic AI and engineering research and should be systematically integrated into regulatory practice.
- AI Trustworthiness principles must be translated into measurable and testable properties, including robustness in edge cases, explainability, and effective human oversight. This requires systematic experimentation under representative operational conditions and in a scientifically rigorous and transparent manner.
- Harmonized testing standards should be established through shared evidence and expertise to ensure comparability and transferability across sites and jurisdictions. Coordinated efforts between regulators, industry, and academia are needed to define common performance metrics, protocols, and evidence requirements for advanced operations. Academic expertise can facilitating joint evaluation of AI systems, analyzing shared datasets, and translating experimental results into guidance that supports regulatory convergence.
- Controlled testing in scientifically-grounded sandbox environments like LINA should be expanded to allow researchers, industry and regulators to jointly explore new operational concepts. Such environments enable informed risk-taking, accelerate regulatory learning, and reduce uncertainty around acceptable risk and mitigation strategies. They should support experimentation at scale including BVLOS missions, mixed manned–unmanned traffic scenarios, and repeated, risk-scaled experimentation in operationally relevant and realistic environments.

In this context, LINA serves as a pivotal platform for an infrastructure-based approach to unmanned aviation. Building on this foundation, the LINA consortium aims to expand this model by integrating additional partners and specialized sites into a broader Swiss framework, forming the basis of the follow-up initiative TESTAIR. TESTAIR will be a scalable, expert-supported experimentation ecosystem enabling harmonized testing, continuous assurance, and long-term capability building in physical AI and aerial robotics, and for broad applications including long-range BVLOS operations. By addressing structural bottlenecks at the intersection of technology, regulation, and infrastructure, this approach provides a concrete pathway toward the safe, certifiable, and societally acceptable deployment of autonomous aerial systems in Switzerland and Europe.

## Data Availability

The original contributions presented in the study are included in the article/supplementary material, further inquiries can be directed to the corresponding author.

## Footnotes

1
https://www.lina.swiss/
